# Sex Modifies the Impact of Type 2 Diabetes Mellitus on the Murine Whole Brain Metabolome

**DOI:** 10.3390/metabo13091012

**Published:** 2023-09-14

**Authors:** Jennifer E. Norman, Saivageethi Nuthikattu, Dragan Milenkovic, Amparo C. Villablanca

**Affiliations:** 1 Division of Cardiovascular Medicine, Department of Internal Medicine, University of California, Davis. 1 Shields Ave, Davis, CA 95616, USA; snuthikattu@ucdavis.edu (S.N.); avillablanca@ucdavis.edu (A.C.V.); 2 Department of Nutrition, University of California, Davis. 1 Shields Ave, Davis, CA 95616, USA; dmilenkovic@ucdavis.edu

**Keywords:** type 2 diabetes mellitus, dementia, brain, metabolomics, sex differences

## Abstract

Type 2 diabetes mellitus (T2DM) leads to the development of cardiovascular diseases, cognitive impairment, and dementia. There are sex differences in the presentation of T2DM and its associated complications. We sought to determine the impact of sex and T2DM on the brain metabolome to gain insights into the underlying mechanisms of T2DM-associated cognitive complications. Untargeted metabolomic analysis was performed, using liquid chromatography-mass spectrometry, on whole brain tissue from adult male and female *db*/*db* mice (a T2DM model) compared to wild-type (WT) C57Bl6/J mice. Regardless of sex, T2DM increased free fatty acids and decreased acylcarnitines in the brain. Sex impacted the number (103 versus 65 in males and females, respectively), and types of metabolites shifted by T2DM. Many choline-containing phospholipids were decreased by T2DM in males. Female-specific T2DM effects included changes in neuromodulatory metabolites (γ-aminobutyric acid, 2-linoleoyl glycerol, *N*-methylaspartic acid, and taurine). Further, there were more significantly different metabolites between sexes in the T2DM condition as compared to the WT controls (54 vs. 15 in T2DM and WT, respectively). T2DM alters the murine brain metabolome in both sex-independent and sex-dependent manners. This work extends our understanding of brain metabolic sex differences in T2DM, cognitive implications, and potential sex-specific metabolic therapeutic targets.

## 1. Introduction

Type 2 diabetes mellitus (T2DM) is a major contributor to the development of cardiovascular diseases (CVD), and increases the risk of developing dementia and cognitive impairment [1,2]. Brain metabolic changes in individuals with T2DM include changes in energy metabolism, lipid membrane metabolism, and neurotransmission [3]. Further, T2DM may contribute to abnormal neuronal activity through endothelial dysfunction-induced breakdown of the blood–brain barrier caused by toxic accumulation of lipids, advanced glycation end-products, and protein aggregates [4]. Thus, a thorough analysis of the brain metabolome in T2DM may help further understand the connection between T2DM and cognitive decline.

There are sex differences in both the presentation and associated complications of T2DM. Men are more likely to be diagnosed with T2DM at an earlier age, but women with T2DM have a greater mortality rate and a greater burden of complications, including a higher risk for CVD, cognitive impairment, and vascular dementia [1,5,6]. Additionally, sex is an important biologic variable affecting the brain, with sex differences seen in cognition as well as the incidence and presentation of neurological diseases [7]. Further, sex differences have been found in brain glycolysis and mitochondrial metabolism [8,9]. Thus, it is plausible that sex differences in T2DM-induced cognitive complications could occur due to underlying metabolic differences. Sex differences are evident in the brain metabolome of mouse models of type 1 diabetes mellitus (T1DM) [10,11,12]. In addition, studies have demonstrated changes in the brain metabolome in the context of T2DM; however, these studies have not addressed sex as a variable [13,14,15]. Thus, there is a gap in knowledge with regards to the impact of sex in the brain metabolome in the context of T2DM.

The *db*/*db* mouse model of T2DM is the most commonly used animal model for T2DM pharmacotherapy [16]. The model is the result of a single mutation leading to a defective leptin receptor. Leptin is a hormone released from adipose tissue that is important in the regulation of metabolism [17]. The *db*/*db* genotype results in obesity and elevated levels of blood glucose, insulin, and cholesterol [16]. In these mice, hyperinsulinemia is seen starting at approximately 2 weeks of age, obesity at 3–4 weeks of age, and hyperglycemia at 4–8 weeks of age [18]. In addition, adult *db*/*db* mice exhibit cognitive dysfunction at 3–5 months of age [19,20]. Therefore, the *db*/*db* mouse model is suitable for investigating the impact of T2DM on the brain metabolome.

In this study, we sought to comprehensively determine the impact of T2DM on the brain metabolome, with our primary outcome being the metabolomics of whole brain tissue in the *db*/*db* mouse model as compared to control wild-type (WT) mice. We examined both males and females separately to identify if there were sex differences in the response of the brain metabolome in T2DM. We hypothesized that *db*/*db* mice would exhibit an altered brain metabolome, with shifts in glucose and lipid metabolism, and that sex would modify the response of the brain metabolic profile to T2DM.

## 2. Materials and Methods

### 2.1. Animals

Research was conducted in conformity with the Public Health Service Policy on Humane Care and Use of Laboratory Animals and reporting was performed in compliance with the ARRIVE guidelines [21]. Food intake, water intake, and activity were monitored daily by vivarium staff to ensure the well-being of the animals. Euthanasia was established as a humane endpoint. For all procedures, care was taken to reduce any discomfort to the animals. No adverse events occurred due to the procedures in this study. A protocol detailing the research question, all procedures, and planned analyses was prepared prior to the study and approved by the Institutional Animal Care and Use Committee of the University of California, Davis (protocol number 22598, initial approval: 14 December 2021; subsequent review and reapproval: 1 December 2022). For all animals the procedures described below were carried out in the same order and at the same timepoints relative to mouse age to minimize confounders.

Male and female T2DM model *db*/*db* (stock number 000697, Jackson Laboratories, Bar Harbor, ME, USA) and control WT (stock 000664, Jackson Laboratories, Bar Harbor, ME, USA) mice were received at 10 weeks of age (4 groups: *db*/*db* males, *db*/*db* females, WT males, and WT females, *n* = 10 mice per group). From receipt, all mice were fed the AIN-93M diet [22] (catalog number, TD.00102 Envigo Teklad diets, Madison, WI, USA) ad libitum; the macronutrient composition of the diet was 4.1% fat, 68.3% carbohydrate, and 12.4% protein (*w*/*w*), with an energy content of 3.6 kcal/g. Animals were housed individually in duplex cages in a temperature-controlled and humidity-controlled environment with a 12 h light/dark cycle in the University of California, Davis Mouse Biology Program. At 17 weeks of age, mice underwent a glucose tolerance test (GTT) and assessment of fasting glucose levels. At 18 weeks of age, mice were transferred to the investigators’ laboratory and allowed to acclimatize for at least one hour prior to euthanasia. Mice were fasted beginning 8 h prior to the time of euthanasia. Blood and whole brain tissue were collected for assessment of fasting serum insulin, total cholesterol, and brain metabolomics. We selected 18 weeks of age (4.5 months) for our terminal timepoint. Others have shown that *db*/*db* mice will exhibit poorer performance on cognitive tests by this time frame (3–5 months of age) as compared to WT mice [19]. A flowchart of the experimental procedures can be found in Figure S1. For all endpoints, the experimental unit was one mouse, or a sample obtained from one mouse. The number of animals or samples used for each endpoint and details of the procedures are detailed in the following sections.

### 2.2. Peripheral Metabolic Assessment

For measurement of fasting serum glucose and glucose tolerance, mice (*n* = 8 per group) were fasted for 8 h, then baseline blood (approximately 5 µL) samples were collected from a tail tip cut onto a glucose test strip. Following the initial blood sampling, a glucose tolerance test (GTT) was performed by injecting a 20% glucose solution intraperitoneally at a dose of 2 g/kg bodyweight. Blood samples for determination of glucose levels were then taken through the initial tail tip cut onto glucose test strips 15, 30, 60, and 120 min after glucose injection. Blood glucose from all samples was determined using Accu-Chek Aviva meter with Accu-Chek Aviva plus test strips (Roche, Basel, Switzerland).

After an 8 h fast at the time of euthanasia, blood samples for the measurement of fasting insulin and cholesterol levels were obtained by ventricular puncture. This was done in the surgical plane of anesthesia to ensure no discomfort or suffering. Serum was separated from whole blood by centrifugation and stored at −80 °C until assayed. Total cholesterol was measured using enzymatic assays from Fisher Diagnostics (Middleton, VA, USA). Insulin was determined by electrochemiluminescence from Meso Scale Discovery (Rockville, MD, USA). Assays were performed in triplicate by the University of California, Davis Metabolic Phenotyping of Mouse Models of Obesity and Diabetes Center. Total cholesterol and insulin were measured on serum from *n* = 9–10 samples per group. Insufficient blood was collected from one *db*/*db* female, leaving this group with *n* = 9 samples; all other groups had *n* = 10 samples for insulin and cholesterol analyses.

### 2.3. Metabolomics Tissue Preparation

Whole brain tissue was collected for metabolomic analyses from *n* = 6 mice per group. This sample size was chosen for the metabolomics endpoint as it was previously demonstrated to be sufficient for identifying brain metabolomic differences induced by T1DM and sex [11]. The brain was collected as quickly as possible following euthanasia, snap-frozen in liquid nitrogen, and stored at −80 °C. The tissue was then powdered while taking care to keep frozen (all tools and samples kept on dry ice). Powdered tissue was kept frozen at −80 °C (or on dry ice during transport) until analyses were completed.

Untargeted metabolomic analysis was performed by Creative Proteomics. Although blinding was not possible for most of the study due to obvious phenotypic differences, Creative Proteomics was blinded as to the group allocation of the samples they received. Untargeted metabolomic analysis was performed as follows: 50 milligrams of tissue was combined with 800 µL of 80% methanol in a tube. The mixture was vortexed for 30 s, homogenized for 90 s by adding two 5-mm metal balls to the tube and using a MM 400 mill mixer at 30 Hz, then sonicated for 30 min at 4 °C. Samples were then kept at −20 °C for one hour, vortexed for 30 s, and kept at 4 °C for 30 min. After centrifugation, 200 µL of supernatant was combined with 5 µL of 0.5 mg/mL DL-o-Chlorophenylalanine and transferred to a vial for liquid chromatography-mass spectrometry (LC-MS) analysis.

### 2.4. LC-MS Analysis

The LC-MS system consisted of ACQUITY UPLC HSS T3 (100 × 2.1 mm × 1.8 μm) with ACQUITY UPLC (Waters, Milford, MA, USA) combined with Q Exactive MS (Thermo Fisher Scientific, Waltham, MA, USA) and screened with electrospray ionization (ESI) mass spectrometry. The mobile phase was composed of solvent A (0.05% formic acid water) and solvent B (acetonitrile) with a gradient elution (0–1 min, 5% B; 1–12 min, 5–95% B; 12–13.5 min, 95% B; 13.5–13.6 min, 95–5% B; 13.6–16 min, 5% B). The flow rate of the mobile phase was 0.3 mL·min^−1^. The column temperature was maintained at 40 °C, and the sample manager temperature was set at 4 °C. Mass spectrometry parameters are shown in Table S1.

Peak areas were normalized based on the total ion count (TIC) method, the most common and simple approach to normalization of LC-MS data [23]. An example calculation is shown here:

normalized data (peak 1) = rawdata (peak 1)/sample (total peak area) × 1,000,000

This was done for each ESI mode separately. The same amount of extract was obtained from each sample and mixed as quality control (QC) samples. The QC samples were prepared using the same sample preparation procedure. DL-o-Chlorophenylalanine was used as an internal standard in this assay.

### 2.5. Pathway Analysis

We conducted pathway overrepresentation analyses of the significantly changed metabolites utilizing the freely available IMPaLA software [24,25,26]. We used the Kyoto Encyclopedia of Genes and Genomes (KEGG) identifiers for all metabolites that could be found to run the pathway analysis in the program.

### 2.6. Statistical Analysis

No animals or obtained samples were removed from analyses, although insufficient blood volume was obtained for serum analyses of insulin and cholesterol for one animal, as detailed in Section 2.2.

Statistical analysis for body weight, GTT, and serum parameters was conducted using Prism (GraphPad Software, San Diego, CA, USA). To test the data for outliers, we used the ROUT (Q-1%) outlier test. No outliers were identified or removed. If the data were normally distributed as determined by the Kolmogorov–Smirnov test, groups were compared by *t*-test (with Welch’s correction if equal variance assumption was not met). For data that were not normally distributed, the Kolmogorov–Smirnov test was used. Differences were considered statistically significant if *p* < 0.05.

Analysis of the untargeted metabolomics data was conducted using MetaboAnalyst 5.0 [27,28]. Normalized peak intensity data was provided by Creative Proteomics. No filtering was applied, and data was autoscaled prior to analyses in MetaboAnalyst. Hierarchical clustering heatmaps of all measured metabolites were made using MetaboAnalyst with Pearson as a distance measure and the Ward clustering method. For pairwise comparisons, parametric *t*-tests were used. Differences were considered statistically significant if the adjusted *p* < 0.05. Venn diagrams were generated using BioVenn [29]. Heatmaps of specific subsets of metabolites were created using Morpheus [30] with one minus Pearson correlation metric and average linkage method.

## 3. Results

### 3.1. Confirmation of Hyperglycemic, Hyperinsulinemic, and Hypercholesterolemic db/db Animal Model

As expected, body weight and fasting levels of insulin, glucose, and cholesterol were all significantly (*p* < 0.05) increased in both male and female *db*/*db* mice as compared to sex-matched WT mice. In addition, the area under the curve (AUC) for a 2-h GTT increased in *db*/*db* mice as compared to sex-matched WT mice (Table 1).

### 3.2. Global Metabolic Profiles of the Brain Metabolome

**Figure 1:**
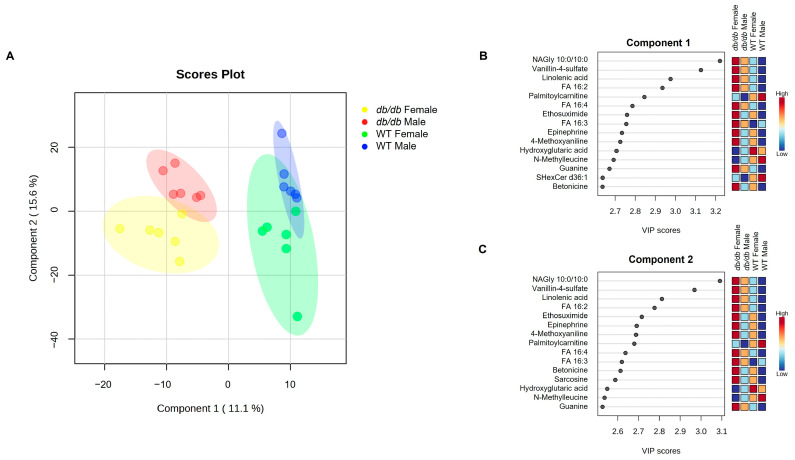
PLS-DA comparisons of brain metabolites in response to T2DM in male and female mice. (**A**) PLS-DA scores plot of metabolites from male and female T2DM *db*/*db* mice, compared to control WT mice. Individual mouse profiles are represented with a small colored circle, while the larger shaded regions of the same color indicate the 95% confidence interval of the group. VIP scores are shown for the top 15 variables for component 1 (**B**), and component 2 (**C**). *n* = 6 per group.

**Figure 2:**
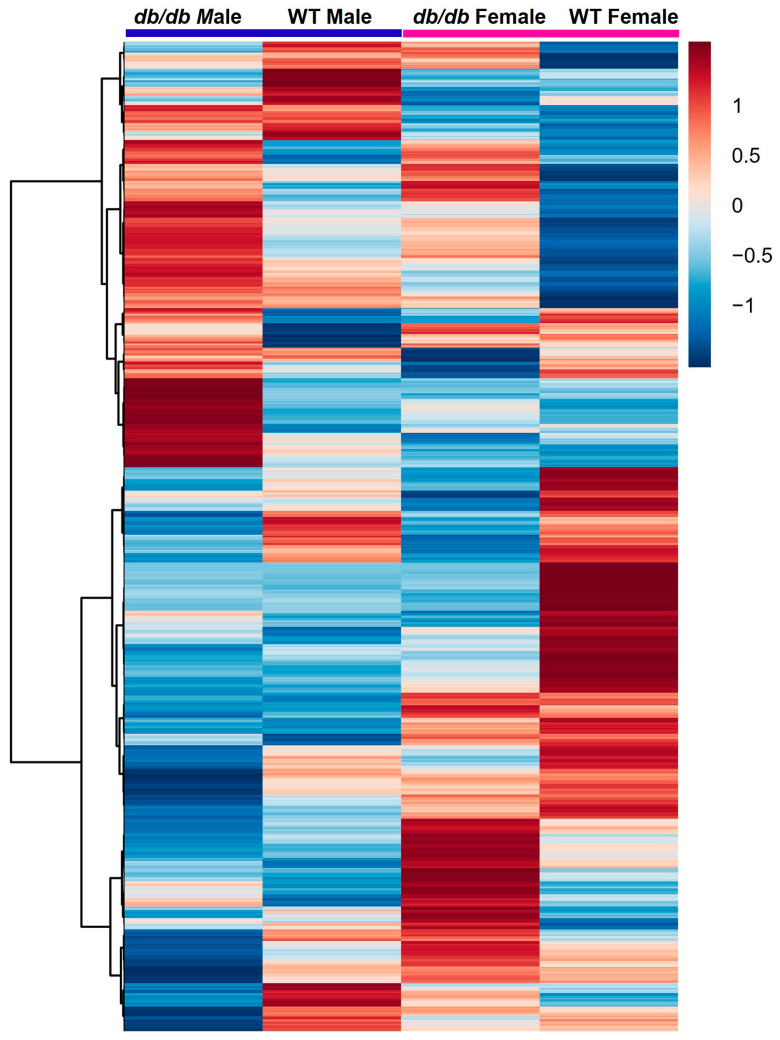
Heatmap of group averages with hierarchical clustering of brain metabolites in response to T2DM in male and female mice. Relative levels of metabolites are indicated by a color scale; darker shades of blue indicate lower levels of a metabolite, while darker shades of red indicate higher levels of a metabolite.

A total of 1021 different metabolites were identified and included in analyses. To determine if there were differences in the global metabolic profiles of the four groups studied (*db*/*db* males, *db*/*db* females, WT males, and WT females), we compared their metabolic profiles using partial least squares-discriminant analysis (PLS-DA). As shown in Figure 1A, *db*/*db* mice were completely segregated from WT mice, regardless of sex. However, there was some overlap of males and females of the same genotype. Variable importance projection (VIP) scores for components 1 and 2 identified the top 15 metabolites accounting for the PLS-DA clustering. They are listed in descending order by scores for component 1 (Figure 1B) and component 2 (Figure 1C). To visualize the magnitude of the differences between groups, a heatmap was constructed of the group averages of brain metabolites (Figure 2). This analysis demonstrated and confirmed that, compared to WT mice, *db*/*db* mice exhibited distinctly different brain metabolite patterns.

We next conducted pairwise analyses and further examined the metabolites that differed between groups, first analyzing the results by diabetes status (Section 3.3), and then by sex (Section 3.4).

### 3.3. Effect of T2DM on the Brain Metabolome

#### 3.3.1. Metabolites Altered by T2DM

We conducted pairwise comparisons of metabolites between *db*/*db* and WT mice for males and females separately. To visually identify the differences in the brain metabolome, we constructed volcano plots of these comparisons. As detailed below, more metabolites were increased than decreased in *db*/*db* mice as compared to WT mice. In addition, fewer metabolites differed between *db*/*db* mice and WT in females compared to males.

**Figure 3:**
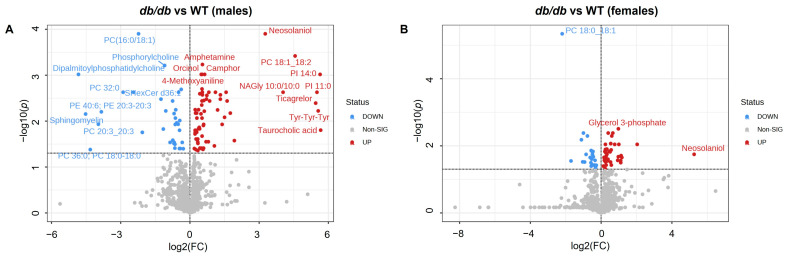
Effects of T2DM on the brain metabolome. Volcano plot of metabolites in T2DM *db*/*db* versus control WT for male (**A**) and female (**B**) mice. No fold change cutoff was applied. All metabolites indicated in blue (downregulated) and red (upregulated) were significant as determined by adjusted *p* < 0.05. *n* = 6 per group.

T2DM significantly altered (adjusted *p* < 0.05) 103 metabolites between male *db*/*db* and male WT mice (Figure 3A). Of these, 67 metabolites were increased in male *db*/*db* mice, with 19 of these being increased more than 2-fold vs. WT. In male *db*/*db* mice, 36 metabolites were decreased, with 14 of these being less than 0.5-fold vs. WT. A complete list of all metabolites that were significantly different between male *db*/*db* and male WT mice, along with their fold change and adjusted *p*-values, can be found in Table S2.

T2DM significantly altered (adjusted *p* < 0.05) 65 metabolites between female *db*/*db* and female WT mice (Figure 3B). Of these, 40 metabolites were increased in female *db*/*db* mice, with 7 of these being more than 2-fold vs. WT. In female *db*/*db* mice, 25 metabolites were decreased, with 5 of these being less than 0.5-fold of the WT levels. A complete list of all metabolites that were significantly different between female *db*/*db* and female WT mice, along with their fold changes and adjusted *p*-values, can be found in Table S3.

**Figure 4:**
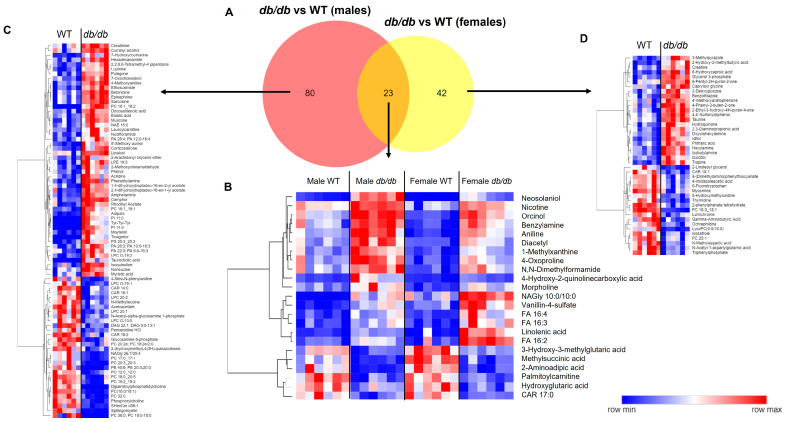
Comparison of the brain metabolic response to T2DM in male and female mice. (**A**) Venn diagram comparison of significant metabolites in T2DM *db*/*db* vs. control WT male and female mice. Heatmaps of significant T2DM-altered metabolites in common between male and female mice (**B**), metabolites that were male-specific (**C**), and metabolites that were female-specific (**D**). In heatmaps, relative levels of metabolites are indicated by a color scale; darker shades of blue indicate lower levels of a metabolite, while darker shades of red indicate higher levels of a metabolite.

For our next step, we compared the metabolites significantly altered by T2DM. T2DM altered 80 metabolites in a male-specific manner and 42 metabolites in a female-specific manner. Only 23 metabolites were altered by T2DM in common in both males and females (Figure 4A). For these 23 metabolites, the direction of change was the same in both sexes, with 6 metabolites decreased and the remaining 17 metabolites increased in *db*/*db* as compared to WT mice (Figure 4B). Heatmaps of the sex-specific metabolites altered by T2DM are provided in Figure 4C for males and Figure 4D for females.

#### 3.3.2. Metabolite Classes and Pathways Shifted by T2DM

**Figure 5:**
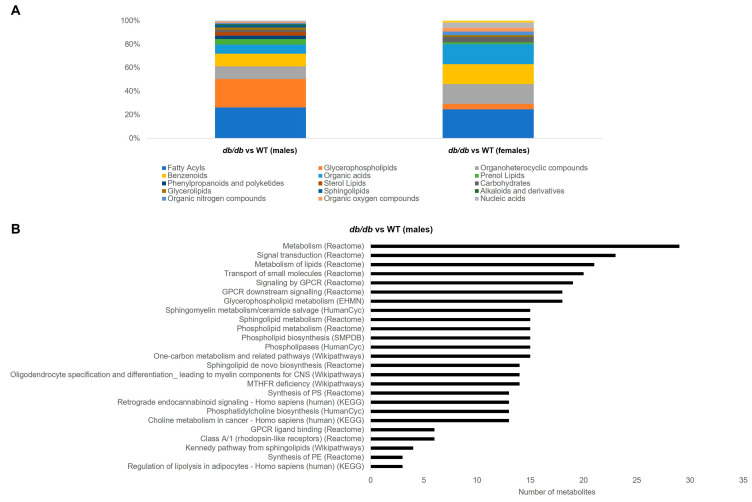
Classification and pathway overrepresentation analysis of brain metabolites altered by T2DM. (**A**) Classifications of metabolites significantly altered by T2DM (*db*/*db* versus WT) in male and female mice. (**B**) All significant (*Q* < 0.05) pathways for metabolites altered by T2DM (*db*/*db* versus WT) in male mice (*y*-axis), and number of metabolites in each pathway (*x*-axis).

The largest category of metabolites that was changed by T2DM, irrespective of sex, was fatty acyls. This category made up 25–26% of significantly different metabolites in T2DM mice. The next 3 most numerous categories of brain metabolites affected by T2DM in male mice were: glycerophospholipids (24%), followed by organoheterocyclic compounds (11%), and benzenoids (11%). In contrast, in T2DM female mice, the next three most numerous categories were equally distributed between organoheterocyclic compounds, benzenoids, and organic acids, each comprising 11% of the significantly different metabolites. Figure 5A summarizes the categories of the metabolites altered by T2DM.

We next conducted pathway overrepresentation analysis of the metabolites altered by T2DM. The metabolites altered by T2DM in male mice significantly (*Q* < 0.05) overrepresented 25 pathways. These pathways were related to metabolism of lipids, including phospholipids and sphingolipids, G-protein-coupled receptor (GPCR) signaling, and one carbon metabolism (Figure 5B). In contrast, the metabolites altered by T2DM in female mice did not exhibit any significantly overrepresented pathways.

### 3.4. Effect of Sex on the Brain Metabolome

#### 3.4.1. Metabolites Altered by Sex

**Figure 6:**
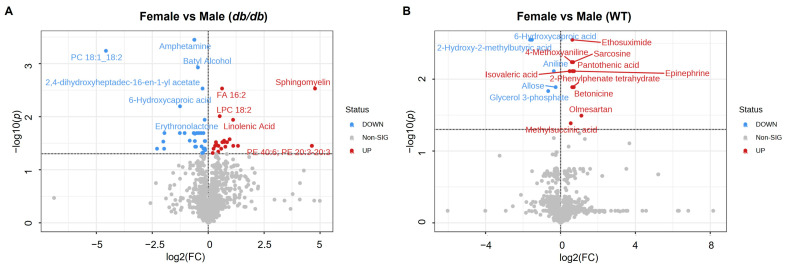
The impact of female versus male sex on brain metabolites. Volcano plot of brain metabolites in female compared to male T2DM *db*/*db* (**A**) and control WT (**B**) mice. No fold change cutoff was applied. All metabolites in blue (downregulated) and red (upregulated) were significant as determined by adjusted *p* < 0.05. *n* = 6 per group.

We examined the effect of sex on the metabolites of the brain in *db*/*db* and WT mice. A total of 54 metabolites differed significantly (adjusted *p* < 0.05) between female *db*/*db* and male *db*/*db* mice (Figure 6A), compared to only 15 metabolites that differed significantly between female WT and male WT mice (Figure 6B). A greater proportion of the metabolites that differed by sex in *db*/*db* mice were lower in females as compared to males (32 lower in females and 22 higher in females). In contrast, within WT mice a larger proportion of the metabolites differing by sex were higher in females compared to males (10 metabolites higher in females and 5 metabolites lower in males).

A complete list of metabolites that were significantly different between females and males, along with the fold change and adjusted *p*-values, can be found in Table S4 for *db*/*db* mice, and Table S5 for WT mice.

#### 3.4.2. Comparison of Metabolites Altered by Sex in T2DM and Control Mice

**Figure 7:**
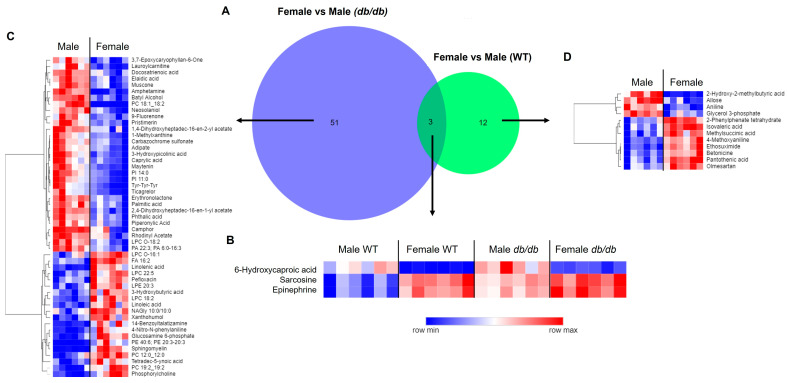
Comparison of brain metabolic sex differences in T2DM and control mice. (**A**) Venn diagram comparison of metabolites significantly different by sex in T2DM *db*/*db* compared to control WT mice. Heatmaps of metabolites exhibiting sex differences common to T2DM *db*/*db* and control WT mice (**B**); in *db*/*db* mice, but not in WT mice (**C**); and in WT mice, but not in *db*/*db* mice (**D**). In heatmaps, relative levels of metabolites are indicated by a color scale; darker shades of blue indicate lower levels of a metabolite, while darker shades of red indicate higher levels of a metabolite.

A larger number of metabolites were altered by sex in T2DM mice than in control mice. A total of 51 metabolites were altered by sex unique to *db*/*db* mice, as compared to 12 metabolites altered by sex unique to WT mice (Figure 7A). Only three brain metabolites differed between sexes common to both *db*/*db* and WT mice, with females having lower levels of 6-hydroxycaproic acid, and higher levels of sarcosine and epinephrine than males (Figure 7B). Heatmaps of the specific metabolites modified by sex for each genotype are provided in Figure 7C and Figure 7D for *db*/*db* and WT mice, respectively.

#### 3.4.3. Metabolite Classes and Pathways Altered by Sex

**Figure 8:**
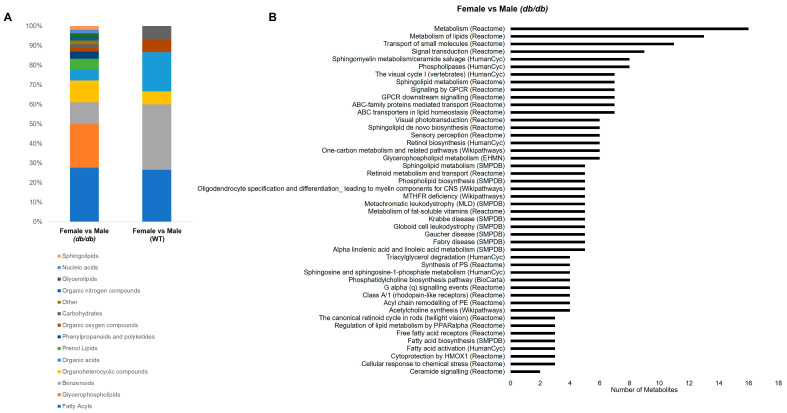
Classification and pathway overrepresentation analysis of brain metabolites altered by sex. (**A**) Classifications of metabolites significantly different by sex in T2DM *db*/*db* and control WT mice. (**B**) All significant (*Q* < 0.05) overrepresented pathways for metabolites altered by sex in T2DM *db*/*db* mice (*y*-axis), and number of metabolites in each pathway (*x*-axis).

Figure 8A shows the categories of all the metabolites that significantly differed by sex in *db*/*db* and WT mice. The metabolites that differed by sex in *db*/*db* mice were mostly fatty acyls (28%), followed by glycerophospholipids (22%), benzenoids (11%), and organoheterocyclic compounds (11%). The few metabolites that differed by sex in WT mice were mostly benzenoids (33%), followed by fatty acyls (27%) and organic acids (20%). Glycerophospholipids were the second most numerous category of metabolites altered by sex in *db*/*db* mice, but were not altered by sex in WT mice. Thus, in addition to the greater number of metabolites that differed by sex in T2DM as compared to the control, the categories of metabolites altered by sex were also shifted.

The most overrepresented pathways affected by sex in *db*/*db* mice were related to lipid metabolism, primarily sphingolipid, phospholipid, and fatty acid metabolism. Additionally, GPCR signaling and one carbon metabolism were represented by pathways altered by sex. Figure 8B shows a complete list of the significantly overrepresented pathways in the metabolites altered by sex in *db*/*db* mice. There were no significant overrepresented pathways in the metabolites differing by sex in WT mice, likely due to the low number of metabolites that differed between males and females in WT mice.

## 4. Discussion

To our knowledge, this is the first study to address sex differences in the whole brain metabolome in response to a T2DM model. We demonstrated that both T2DM and sex alter the global brain metabolomic profile in profound ways. We discuss our findings both in terms of the sex-independent and sex-dependent effects of T2DM on the brain metabolome. Our findings are further contextualized in terms of implications of T2DM in dementia.

### 4.1. Sex-Independent Consequences of T2DM on the Brain Metabolome

Our study indicated that irrespective of sex, fatty acyls were the predominant category of metabolites altered by T2DM. Free fatty acids and acylcarnitines were the most prominent fatty acyl subgroups altered. Furthermore, T2DM increased four long-chain free fatty acids (FA 16:2, FA 16:3, FA 16:4, and linolenic acid) in both males and females. β-oxidation comprises roughly 20% of energy metabolism in the brain, and the accumulation of free fatty acids is believed to be toxic [31,32]. Fatty acids can also regulate membrane fluidity and permeability, function as second messengers, and play a role in synaptic vesicle recycling [31]. As fatty acid metabolism is dysregulated in the brains of individuals with Alzheimer’s disease (AD) [33], this could be a potential mechanism of T2DM-induced cognitive decline.

Two acylcarnitines (CAR 17:0 and palmitoylcarnitine/CAR16:0) were decreased by T2DM, regardless of sex. An acylcarnitine is the product of L-carnitine esterification to a fatty acid, which allows for transport of the fatty acid into the mitochondria [34]. The activity of carnitine palmitoyl transferase, which transports long-chain acylcarnitines into the mitochondria, is lower in the brain than in the periphery. Palmitoylcarnitine may have additional roles in the brain, such as changing membrane fluidity and charge, which can impact the activity of membrane enzymes and transporters [35]. Compared to healthy individuals, serum levels of acylcarnitines are reduced in both individuals with mild cognitive impairment and those with AD [36]. Additionally, plasma levels of acylcarnitines are negatively correlated with processing speed [37].

Therefore, the increase in fatty acids, coupled with the decrease in acylcarnitines, in the brains of T2DM mice could be an indicator of reduced β-oxidation. Further, these changes could have implications for reduced cognitive function through lipotoxicity and alterations in important physiological processes, including cell membrane fluidity and permeability.

### 4.2. Sex-Dependent Consequences of T2DM on the Brain Metabolome

Our study showed that most of the brain metabolic effects of T2DM were sex-dependent. In addition, the number of brain metabolites that differed, and the magnitude of the difference between T2DM and control mice, was greater in males than females. Furthermore, more than three times the number of metabolites differed by sex in T2DM mice compared to control mice.

#### 4.2.1. Sex-Dependent Effects of T2DM in Males

In males, 80 of the 103 metabolites that were significantly altered by T2DM were specific to males. Glycerophospholipids constituted nearly one quarter of metabolites altered by T2DM in males; this category was highlighted by both classification and pathway analysis. In contrast, only three glycerophospholipids were altered by T2DM in females. Additionally, glycerophospholipids constituted 22% of the metabolites that differed between male and female T2DM mice. No glycerophospholipids differed between the sexes in control WT mice. This suggests that T2DM alters glycerophospholipids in a primarily male-specific manner.

Glycerophospholipids are the primary components of cell membranes, modulating stability, fluidity, and permeability. These functions are central to the proper functioning of membrane proteins, including receptors and ion channels [31]. Shifts in glycerophospholipids accompany AD pathology in mouse models and postmortem human brain tissue [38,39,40]. Of the glycerophospholipids altered by T2DM in our study, most were choline-containing. Choline-containing phospholipids can modulate the physiology of the neurovascular unit and a loss of these phospholipids can contribute to neurodegenerative diseases such as AD; lower levels are also associated with greater severity of AD pathology [40,41]. Most of the choline-containing phospholipids altered by T2DM were decreased. Many of these were phosphatidylcholines, the main phospholipid of the outer layer of cell membranes in mammals, which accounts for 32.8% of the total glycerophospholipid content of the human brain [41]. Thus, in males, the reduction in choline-containing phospholipids may be a primary contributor to T2DM-induced neurodegeneration and detrimental effects on cognitive function.

#### 4.2.2. Sex-Dependent Effects of T2DM in Females

Of the 65 metabolites altered by T2DM in females, 42 were specific to females. Unlike in males, there was not a large female-specific category of T2DM-shifted metabolites or enriched pathways. However, there were female-specific T2DM-altered metabolites with important neuromodulator functions. T2DM in females decreased brain levels of γ-aminobutyric acid (GABA), 2-linoleoyl glycerol, and *N*-methylaspartic acid (NMDA), while increasing levels of taurine, with implications as discussed below.

GABA is the primary inhibitory neurotransmitter in the brain; dysfunction of its signaling leads to excessive neuronal activity, which may contribute to cognitive impairment [42,43]. Further, GABA levels are reduced in the brains of AD rodent models and humans with AD [44,45,46,47]. The monoacylglycerol 2-Linoleoyl glycerol has been shown to be a partial agonist of the cannabinoid type 1 receptor (CB1) [48]. CB1 agonists can prevent excitotoxicity (toxic actions of excessive excitatory signaling) [49]. Therefore, a reduction in 2-linoleoyl glycerol could contribute to excitotoxicity in the brain. NMDA is primarily present in the nervous and endocrine tissues [50], and acts as an agonist for the excitatory amino acid NMDA receptors [51]. NMDA receptors, and dysfunction of transmission of their signals, have been implicated in the development of AD [52,53]. Taurine is an amino acid that is not considered a neurotransmitter, because a specific receptor has not been identified [54]. However, taurine has inhibitory effects through interactions with GABA, glycine, and NMDA receptors [42,43,51,54,55,56,57].

Taken together, our findings suggest that T2DM alters the brain metabolome of females in a manner associated with modulation of both inhibitory and excitatory neuronal signaling, with potential detriments to cognitive function. This appears to be a mechanism unique to females, as these metabolites were not found to be altered by T2DM in males.

### 4.3. Potential Mechanisms of Sex Differences in the Brain Metabolome

In this study, we demonstrated that biological sex affects the whole brain metabolome. The mechanisms behind this could involve direct effects of sex hormones, gonadal sex, chromosomal sex, or a combination of these factors. Theories behind these metabolic sex differences include higher evolutionary pressure for females, X chromosome dosage, and early life programming [58]. Previous studies have demonstrated that both sex hormones and sex chromosomes can impact aspects of peripheral metabolism [59,60,61] as well as brain structure and behavior [62]. Future studies are needed to investigate the contributions of sex hormones, gonadal sex, and chromosomal sex to the brain metabolome in the context of T2DM to further elucidate the mechanisms behind the sex differences we observed.

### 4.4. Relevance to Human Disease

Our findings suggest that T2DM results in significant shifts in the brain metabolome that are dependent on both diabetes and sex. This may have major implications for a sexually dimorphic role of T2DM in cognitive decline. Further consideration of the mechanisms behind metabolic sex differences is warranted, as discussed in Section 4.3. In addition, regardless of sex, T2DM increased fatty acids and decreased acylcarnitines.

The current study uncovered a large T2DM-induced shift in glycerophospholipids, primarily a reduction in choline-containing phospholipids, in males, with smaller changes seen in females. Previous studies have suggested that glycerophospholipid supplemenation reduces age-related cerebral structural decline, with implications for cognitive decline [63]. Further, supplementation with phosphatidylcholines improved Mini Mental State Examination (MMSE) scores of individuals with age-related cognitive disorders [64,65]. Thus, persons with T2DM, particularly males, may see cognitive benefits from supplementation with choline-containing phospholipids. However, this hypothesis remains to be tested.

In females, our data showed that T2DM altered neuromodulatory metabolites. As there were shifts in metabolites participating in both inhibitory and excitatory signaling, it is difficult to draw specific conclusions about the implications for human disease based on the data from the current study alone. However, future studies should tease out the balance of these signaling pathways in T2DM to identify specific treatment targets for the prevention of T2DM-induced cognitive decline in females.

### 4.5. Study Limitations

We recognize that although we have contextualized the findings of our metabolomic study in terms of cognitive decline and dementia, the study does not include cognitive function assessments. However, others utilizing the same models at a similar age to the mice in our study have demonstrated cognitive dysfunction [19,20], providing evidence that the *db*/*db* model is an appropriate model for T2DM-induced cognitive decline. In addition, while our study characterized the metabolome of the whole brain, there may be region-specific changes induced by T2DM that may be further modified by sex. While others have examined brain regional metabolism in T2DM models, they included only male mice or mixed-sex groups without addressing sex as a variable [13,14,15]. Thus, this remains an area for further investigation.

We recognize that mouse models are not completely analogous with human disease. However, obtaining human brain tissue viable for metabolomic analyses is not feasible, given the need for a very short postmortem interval. The *db*/*db* mouse model we used displays the same metabolic dysfunction as T2DM in humans (obesity, hyperglycemia, hyperinsulinemia, and hypercholesterolemia). Therefore, this model is the most commonly used T2DM model in murine studies.

Furthermore, other mechanisms by which T2DM may affect brain metabolism were not addressed in the current study, and may include those that proceed through vascular injury, glucose/lipid toxicity, blood–brain barrier permeability, and neuroinflammation. Direct assessment of these functional consequences was beyond the scope of this study.

## 5. Conclusions

**Figure 9:**
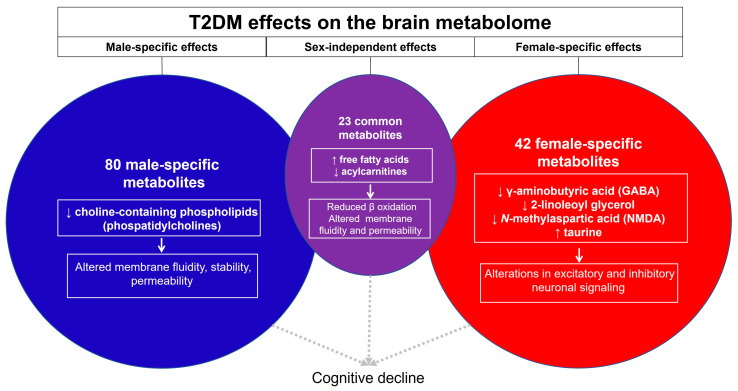
Summary and conceptual understanding of the sex-dependent and sex-independent impact of T2DM on the murine brain metabolome.

While others have described sex differences in the brain metabolomic response to T1DM, we have uncovered novel and important sex differences in the response of the whole brain metabolome to T2DM. In Figure 9, we conceptualize the major findings of our work.

The sex-independent effects of T2DM are driven primarily by an increase in fatty acids and a decrease in acylcarnitines. The implications of this are reduced β-oxidation and dysregulated fatty acid metabolism, potentially leading to lipotoxicity and alterations in second messengers, synaptic vesicle recycling, and membrane fluidity and charge, which can impact membrane enzyme and transporter activity. A large metabolic shift in glycerophospholipids induced by T2DM was seen in males and could contribute to cognitive decline through modulation of cell membrane fluidity, stability, and permeability. In contrast, in females, T2DM appears to modulate both inhibitory and excitatory neuronal signaling through female-specific effects on GABA, 2-linoleol glycerol, NMDA and taurine levels.

Our work extends our understanding of the metabolic mechanisms whereby T2DM alters the brain metabolic profile and has potential implications for how these metabolic alterations may differentially contribute to neurodegenerative processes, including cognitive decline, in males and females. As such, our work may have implications for considering sex-specific targets in addressing T2DM-dependent dysregulation of brain metabolism, with relevance to cognitive decline.

## Figures and Tables

**Table 1:** Fasting serum parameters and GTT.

	Male		Female	
	WT	*db*/*db*	WT	*db*/*db*
Body weight (g)	30.46 ± 2.988	46.78 ± 2.443 *	20.65 ± 1.314	45.62 ± 5.197 *
Insulin (pg/mL)	229.1 ± 131.7	2215 ± 1023 *	182.2 ± 78.72	2197 ± 1350 *
Glucose (mg/dL)	146 ± 33.92	244 ± 72.13 *	129.9 ± 13.72	290.5 ± 134 *
Total Cholesterol (mg/dL)	124.1 ± 35.88	278.1 ± 33.18 *	77.03 ± 17.78	237.7 ± 43.64 *
GTT AUC (mg min/dL)	27,369 ± 6475	56,142 ± 10,998 *	23,465 ± 2475	58,602 ± 11,385 *

Data shown is mean ± standard deviation. * *p* < 0.05 when compared to sex-matched WT mice; *n* = 10 per group (body weight); *n* = 9–10 per group (insulin and total cholesterol); *n* = 8 per group (glucose and GTT AUC).

## Data Availability

Lists of all metabolites which were found to be significantly different for each comparison can be found in the Supplementary Materials. The raw data will be made available upon request.

## Supplementary Materials

The following supporting information can be downloaded at https://www.mdpi.com/article/10.3390/metabo13091012/s1. Figure S1. Flow chart of experimental procedures; Table S1. Mass spectrometry parameters; Table S2. Brain metabolites significantly altered by *db*/*db* genotype in males; Table S3. Brain metabolites significantly altered by *db*/*db* genotype in females; Table S4. Brain metabolites significantly different between female *db*/*db* and male *db*/*db* mice; Table S5. Brain metabolites significantly different between female WT and male WT mice.
