# Supplementary material for: Sex Modifies the Impact of Type 2 Diabetes Mellitus on the Murine Whole Brain Metabolome
Source: Metabolites. 2023 Sep 14;13(9):1012. doi: 10.3390/metabo13091012 (PMC10536706; doi:10.3390/metabo13091012)
Supplement: Supplementary file 1 [file metabolites-13-01012-s001.zip › metabolites-2574354-supplementary.pdf]

## Supplemental Figures

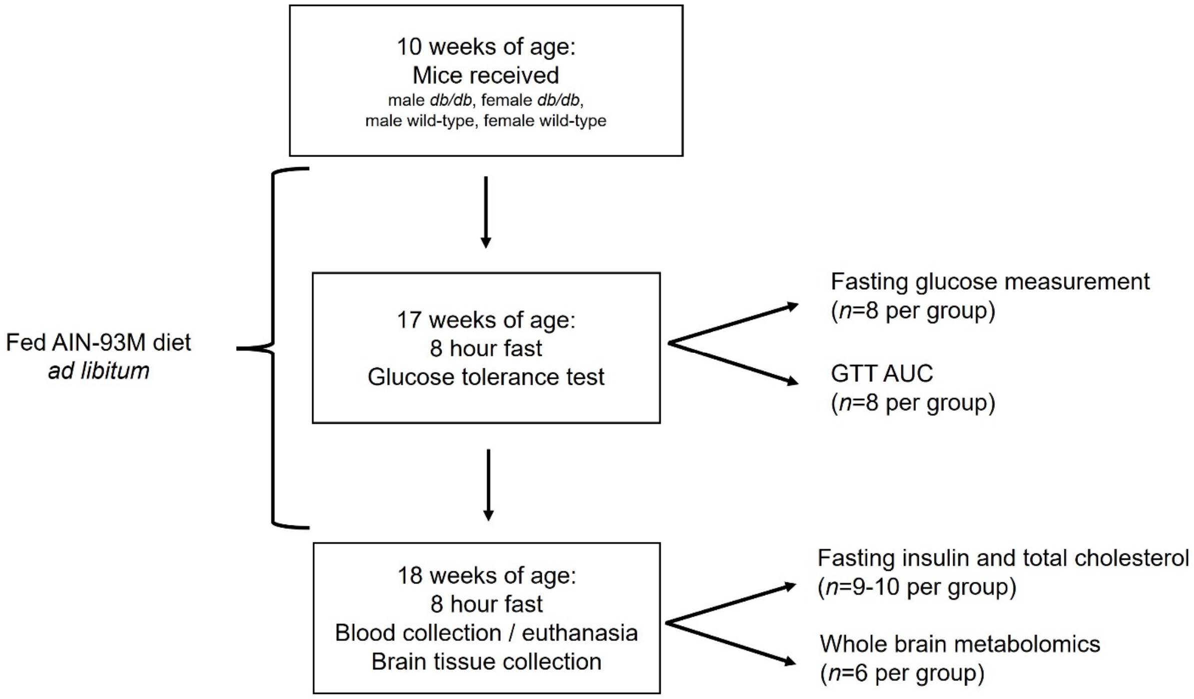


**Figure S1. Flow chart of experimental procedures.**

**Supplemental Tables**

**Table S1. Mass spectrometry parameters.**

| **ESI mode** | **Positive** | **Negative** |
|---|---|---|
| Heater temperature (°C) | 300 | 300 |
| Sheath gas flow rate (arb) | 45 | 45 |
| Auxillary gas flow rate (arb) | 15 | 15 |
| Sweep gas flow rate (arb) | 1 | 1 |
| Spray voltage (kV) | 3.0 | 3.2 |
| Capillary temperature (°C) | 350 | 350 |
| S-Lens radiofrequency level (%) | 30 | 60 |

ESI (electrospray ionization mode)

**Table S2. Brain metabolites significantly altered by *db/db* genotype in males.**

| Metabolite | ESI mode | RT [min] | m/z | FC | log2(FC) | adjusted p-value |
|---|---|---|---|---|---|---|
| Dipalmitoylphosphatidylcholine | + | 11.246 | 734.5676 | 0.035086 | -4.833 | 0.00096 |
| Sphingomyelin | + | 10.921 | 731.6034 | 0.043518 | -4.5223 | 0.007008 |
| PC 36:0; PC 18:0-18:0 | - | 14.502 | 834.6188 | 0.050049 | -4.3205 | 0.041655 |
| PC 20:3_20:3 | + | 14.494 | 834.5971 | 0.063844 | -3.9693 | 0.011688 |
| PE 40:6; PE 20:3-20:3 | + | 14.493 | 792.5511 | 0.069856 | -3.8395 | 0.006273 |
| PC 32:0 | + | 11.672 | 756.5489 | 0.13433 | -2.8962 | 0.00235 |
| SHexCer d36:1 | - | 14.501 | 806.5446 | 0.18295 | -2.4505 | 0.00235 |
| PC(16:0/18:1) | + | 11.649 | 760.5817 | 0.21341 | -2.2283 | 0.000125 |
| PC 17:0_17:1 | + | 14.494 | 760.582 | 0.23909 | -2.0644 | 0.01758 |
| NAGly 26:7/20:4 | + | 14.491 | 759.5716 | 0.23912 | -2.0642 | 0.01758 |
| PC 19:2_19:2 | + | 11.282 | 810.5973 | 0.41885 | -1.2555 | 0.003322 |
| 2-Aminoadipic acid | - | 0.795 | 160.0602 | 0.44941 | -1.1539 | 0.014885 |
| Phosphorylcholine | + | 10.808 | 184.0736 | 0.4676 | -1.0966 | 0.000614 |
| PC 18:0_20:5 | + | 11.656 | 808.579 | 0.4838 | -1.0475 | 0.005993 |
| LPC O-10:0 | + | 10.92 | 398.2685 | 0.5505 | -0.86119 | 0.029106 |
| Glucosamine 6-phosphate | + | 10.93 | 260.0527 | 0.59391 | -0.75168 | 0.003643 |
| 2-(hydroxymethyl)-4(3*H*)-quinazolinone | + | 3.878 | 177.0676 | 0.59775 | -0.74238 | 0.026041 |
| Pentamidine HCl | + | 4.126 | 171.1042 | 0.61758 | -0.6953 | 0.030558 |
| Methylsuccinic acid | - | 2.194 | 131.0336 | 0.62119 | -0.6869 | 0.006853 |
| DAG 22:1; DAG 9:0-13:1 | + | 9.52 | 444.3679 | 0.62328 | -0.68204 | 0.029106 |
| LPC O-16:1 | + | 10.592 | 480.3444 | 0.63564 | -0.65372 | 0.038805 |
| CAR 16:1 | + | 9.006 | 398.326 | 0.63675 | -0.65121 | 0.031875 |
| CAR 14:0 | + | 8.752 | 372.3102 | 0.64262 | -0.63797 | 0.0118 |
| LPC 20:1 | - | 11.772 | 594.377 | 0.646 | -0.63039 | 0.008589 |
| Palmitoylcarnitine | + | 9.595 | 400.3414 | 0.65301 | -0.61483 | 0.005659 |
| 3-Hydroxy-3-methylglutaric acid | - | 1.597 | 161.0443 | 0.67202 | -0.57342 | 0.010942 |
| CAR 18:2 | + | 9.29 | 424.3416 | 0.67283 | -0.57169 | 0.010942 |
| *N*-Methylleucine | - | 9.597 | 144.1015 | 0.70215 | -0.51016 | 0.011688 |
| LPC 20:2 | - | 10.882 | 592.361 | 0.71006 | -0.49399 | 0.00235 |
| CAR 17:0 | + | 10.007 | 414.3572 | 0.72861 | -0.45679 | 0.039581 |
| PC 20:2e; PC 18:2e/2:0 | + | 10.684 | 548.3705 | 0.73131 | -0.45144 | 0.015824 |
| PC 12:0_12:0 | + | 10.175 | 622.4426 | 0.73654 | -0.44116 | 0.009704 |
| Hydroxyglutaric acid | - | 1.233 | 147.0285 | 0.74965 | -0.41572 | 0.039581 |
| Acetoacetate | + | 0.776 | 103.0394 | 0.7693 | -0.37839 | 0.002039 |
| 4-Nitro-*N*-phenylaniline | - | 9.176 | 213.0661 | 0.79324 | -0.33417 | 0.029106 |
| *N*-Acetyl-αglucosamine 1-phosphate | - | 0.897 | 300.0483 | 0.81863 | -0.28871 | 0.039919 |
| Acridine | + | 8.4 | 180.0807 | 1.1414 | 0.19082 | 0.039581 |
| 4-Oxoproline | - | 2.835 | 128.0339 | 1.1662 | 0.22184 | 0.019043 |

| | | | | | | |
|---|---|---|---|---|---|---|
| 2,4-dihydroxyheptadec-16-en-1-yl acetate | + | 10.285 | 311.2577 | 1.1694 | 0.22577 | 0.015824 |
| 1,4-dihydroxyheptadec-16-en-2-yl acetate | + | 10.531 | 311.2577 | 1.1705 | 0.22719 | 0.005642 |
| PA 28:4; PA 12:0-16:4 | - | 10.547 | 583.3354 | 1.172 | 0.22898 | 0.01758 |
| Pulegone | + | 10.691 | 153.1272 | 1.1847 | 0.24447 | 0.006695 |
| Phenol | + | 9.018 | 95.04966 | 1.1959 | 0.25805 | 0.041671 |
| Cuminyl alcohol | + | 10.567 | 133.1011 | 1.2024 | 0.26588 | 0.024581 |
| Myristic acid | - | 12.435 | 227.2009 | 1.2218 | 0.28903 | 0.017319 |
| Rhodinyl Acetate | + | 12.176 | 199.1692 | 1.2232 | 0.2906 | 0.006695 |
| PA 22:3; PA 6:0-16:3 | - | 7.909 | 501.257 | 1.2256 | 0.29346 | 0.017431 |
| PA 26:3; PA 10:0-16:3 | - | 10.2 | 557.3198 | 1.2321 | 0.3011 | 0.008589 |
| Creatinine | - | 1.322 | 112.0501 | 1.2642 | 0.33823 | 0.024581 |
| *N*,*N*-Dimethylformamide | + | 4.177 | 74.0608 | 1.2645 | 0.33861 | 0.028589 |
| Norleucine | + | 1.456 | 132.102 | 1.2675 | 0.342 | 0.043771 |
| 2-Methoxycinnamaldehyde | + | 0.08 | 163.0752 | 1.2708 | 0.34568 | 0.030558 |
| 1-Methylxanthine | + | 5.292 | 167.0553 | 1.289 | 0.36625 | 0.014721 |
| Isoquinoline | + | 3.253 | 130.0653 | 1.2948 | 0.3727 | 0.005642 |
| Benzylamine | + | 0.096 | 108.0811 | 1.3216 | 0.40223 | 0.013696 |
| Diacetyl | + | 0.045 | 87.04469 | 1.3378 | 0.41985 | 0.014721 |
| LPC O-18:2 | + | 11.544 | 506.3601 | 1.3448 | 0.42743 | 0.002537 |
| FA 16:2 | - | 11.976 | 251.201 | 1.3494 | 0.43229 | 0.03866 |
| Aniline | + | 0.092 | 94.06563 | 1.3934 | 0.47862 | 0.00694 |
| Orcinol | + | 0.014 | 125.06 | 1.4043 | 0.48983 | 0.00096 |
| 2-Arachidonyl Glycerol ether | + | 9.865 | 387.2891 | 1.4107 | 0.49641 | 0.039581 |
| Morpholine | + | 15.108 | 88.07627 | 1.4224 | 0.50833 | 0.038805 |
| Epinephrine | + | 2.854 | 184.0969 | 1.426 | 0.51195 | 0.004465 |
| Sarcosine | + | 2.85 | 90.05552 | 1.4356 | 0.52161 | 0.003643 |
| LPE 18:3 | + | 10.149 | 476.2739 | 1.4369 | 0.52299 | 0.008589 |
| Ethosuximide | + | 2.853 | 142.0863 | 1.437 | 0.52306 | 0.001994 |
| Linalool | + | 10.689 | 155.1429 | 1.4398 | 0.52583 | 0.021203 |
| 4-Methoxyaniline | + | 2.852 | 124.0759 | 1.4401 | 0.5262 | 0.00096 |
| Nicotine | + | 9.099 | 163.1228 | 1.4516 | 0.53768 | 0.00235 |
| Elaidic acid | + | 12.135 | 283.2629 | 1.4558 | 0.5418 | 0.002681 |
| Amphetamine | + | 3.25 | 136.1121 | 1.4559 | 0.54194 | 0.000585 |
| NAE 16:0 | + | 12.135 | 300.2895 | 1.4814 | 0.56694 | 0.041236 |
| Hexadecanamide | + | 12.633 | 256.2632 | 1.4979 | 0.58296 | 0.012432 |
| FA 16:3 | - | 11.557 | 249.1854 | 1.507 | 0.59171 | 0.038805 |
| Lauroylcarnitine | - | 11.607 | 342.2642 | 1.5096 | 0.59413 | 0.0118 |
| Betonicine | + | 2.853 | 160.0969 | 1.5149 | 0.59925 | 0.003565 |
| Muscone | + | 12.135 | 239.2369 | 1.5391 | 0.6221 | 0.006082 |
| Camphor | + | 12.882 | 153.1272 | 1.5551 | 0.63702 | 0.00096 |
| Nudifloramide | + | 1.666 | 153.0658 | 1.622 | 0.69775 | 0.028113 |
| Lupinine | + | 12.881 | 170.1538 | 1.6421 | 0.71559 | 0.003322 |

| | | | | | | |
|---|---|---|---|---|---|---|
| 2,2,6,6-Tetramethyl-4 piperidone | + | 12.882 | 156.1382 | 1.6615 | 0.73249 | 0.002751 |
| FA 16:4 | - | 10.874 | 247.1697 | 1.7385 | 0.79787 | 0.028113 |
| Adipate | + | 12.911 | 147.0654 | 1.7653 | 0.8199 | 0.00235 |
| Docosatrienoic acid | - | 14.312 | 333.2797 | 1.8069 | 0.85355 | 0.003643 |
| 4-Hydroxy-2-quinolinecarboxylic acid | + | 11.279 | 190.0498 | 2.084 | 1.0593 | 0.034669 |
| Linolenic acid | - | 12.298 | 277.2168 | 2.1509 | 1.105 | 0.00235 |
| Corticosterone | - | 7.107 | 391.2122 | 2.2956 | 1.1989 | 0.005707 |
| Maytenin | + | 11.444 | 421.271 | 2.4919 | 1.3173 | 0.003322 |
| Vanillin-4-sulfate | - | 4.702 | 230.9962 | 2.4979 | 1.3207 | 0.002681 |
| Phenethylamine | + | 7.208 | 122.0965 | 2.632 | 1.3961 | 0.0118 |
| PC 18:1_18:1 | + | 13.528 | 786.5991 | 2.8299 | 1.5007 | 0.008255 |
| 7-Oxocholesterol | + | 12.453 | 401.3416 | 2.9642 | 1.5677 | 0.00235 |
| 5'-methoxy aureol | + | 7.126 | 329.2107 | 3.0561 | 1.6117 | 0.003643 |
| PS 20:3_20:3 | + | 14.503 | 836.5398 | 3.3632 | 1.7498 | 0.006695 |
| 7-Hydroxycoumarine | + | 6.988 | 163.0389 | 3.7989 | 1.9256 | 0.026569 |
| Neosolaniol | + | 12.627 | 383.1671 | 9.6082 | 3.2643 | 0.000125 |
| NAGly 10:0/10:0 | + | 11.922 | 417.3358 | 16.415 | 4.0369 | 0.00235 |
| PC 18:1_18:2 | + | 11.261 | 784.5834 | 23.565 | 4.5586 | 0.000381 |
| Ticagrelor | + | 10.658 | 523.191 | 43.72 | 5.4502 | 0.004056 |
| PI 11:0 | + | 10.494 | 534.228 | 45.435 | 5.5057 | 0.00235 |
| Tyr-Tyr-Tyr | + | 10.149 | 508.2126 | 47.182 | 5.5602 | 0.005993 |
| PI 14:0 | + | 10.569 | 576.2742 | 49.937 | 5.642 | 0.00096 |
| Taurocholic acid | - | 6.702 | 514.2836 | 50.811 | 5.6671 | 0.015739 |

FC: Fold change.

**Table S3. Brain metabolites significantly altered by *db/db* genotype in females.**

| Metabolite | ESI Mode | RT [min] | m/z | FC | log2(FC) | adjusted p-value |
|---|---|---|---|---|---|---|
| PC 18:0_18:1 | + | 11.638 | 788.6127 | 0.2172 | -2.2029 | 4.58E-06 |
| 2-Linoleoyl glycerol | + | 14.497 | 337.2734 | 0.30721 | -1.7027 | 0.028217 |
| 2-Aminoadipic acid | - | 0.795 | 160.0602 | 0.46148 | -1.1157 | 0.006595 |
| γ-Aminobutyric Acid | + | 1.359 | 104.071 | 0.4856 | -1.0422 | 0.030053 |
| Methylsuccinic acid | - | 2.194 | 131.0336 | 0.49951 | -1.0014 | 0.004157 |
| Thymidine | - | 2.552 | 241.0824 | 0.52379 | -0.93293 | 0.03038 |
| LysoPC(0:0/18:0) | + | 11.543 | 562.3264 | 0.55467 | -0.8503 | 0.017979 |
| 2-Phenylphenate tetrahydrate | + | 4.206 | 171.0803 | 0.5842 | -0.77546 | 0.005078 |
| Hydroxyglutaric acid | - | 1.233 | 147.0285 | 0.64985 | -0.62183 | 0.022241 |
| Ochrephilone | - | 13.672 | 381.173 | 0.66983 | -0.57813 | 0.031932 |
| 3-Hydroxy-3-methylglutaric acid | - | 1.597 | 161.0443 | 0.67619 | -0.5645 | 0.013795 |
| CAR 18:1 | + | 9.802 | 426.3571 | 0.67842 | -0.55976 | 0.040241 |
| 4-(Dimethylamino)phenylthiocyanate | + | 4.206 | 179.0621 | 0.6905 | -0.53429 | 0.024844 |
| 4-imidazoleacetic acid | + | 4.208 | 165.0101 | 0.69359 | -0.52784 | 0.037552 |
| Triphenylphosphate | + | 1.362 | 327.0794 | 0.70315 | -0.5081 | 0.01473 |
| 6-Fluorotryptophan | + | 4.205 | 223.0889 | 0.70603 | -0.5022 | 0.037409 |
| CAR 17:0 | + | 10.007 | 414.3572 | 0.71081 | -0.49246 | 0.0179 |
| Palmitoylcarnitine | + | 9.595 | 400.3414 | 0.72701 | -0.45995 | 0.01473 |
| *N*-Methylaspartic acid | + | 1.354 | 148.0604 | 0.73527 | -0.44366 | 0.037409 |
| *N*-Acetyl-1-aspartylglutamic acid | - | 1.346 | 303.0828 | 0.73645 | -0.44135 | 0.02113 |
| Isosafrole | + | 1.358 | 185.0557 | 0.76231 | -0.39155 | 0.037409 |
| 5-Hydroxymethyluridine | - | 1.51 | 273.0724 | 0.76791 | -0.38099 | 0.037409 |
| Lumichrome | + | 4.012 | 243.0877 | 0.78802 | -0.34369 | 0.027043 |
| Myosmine | + | 4.03 | 147.0918 | 0.80374 | -0.31521 | 0.045278 |
| PC 25:1 | + | 7.363 | 656.4324 | 0.82787 | -0.27252 | 0.037892 |
| 4'-Methoxyacetophenone | + | 8.609 | 151.0752 | 1.0914 | 0.12616 | 0.040241 |
| Phthalic acid | - | 6.413 | 165.0181 | 1.1203 | 0.16385 | 0.022241 |
| 4-Oxoproline | - | 2.835 | 128.0339 | 1.135 | 0.18274 | 0.027043 |
| 2-Ethyl-3-hydroxy-4*H*-pyran-4-one | + | 9.927 | 141.0546 | 1.1581 | 0.21179 | 0.01473 |
| 2-Deoxyglucose | - | 0.09 | 163.0598 | 1.1756 | 0.23336 | 0.048103 |
| Hydroquinone | + | 3.373 | 111.0444 | 1.1786 | 0.23713 | 0.029784 |
| Benzothiazole | + | 6.383 | 136.0215 | 1.1815 | 0.24058 | 0.009049 |
| 4-Phenyl-3-buten-2-one | + | 8.103 | 147.0804 | 1.1828 | 0.24218 | 0.012806 |
| Creatine | + | 0.792 | 132.0768 | 1.1856 | 0.24563 | 0.027043 |
| Dicyclohexylamine | + | 4.828 | 182.1905 | 1.1886 | 0.24921 | 0.030148 |
| Taurine | - | 0.768 | 124.0059 | 1.1923 | 0.25373 | 0.015792 |
| 1-methylxanthine | + | 5.292 | 167.0553 | 1.2118 | 0.27719 | 0.020466 |
| Dulcitol | - | 14.721 | 181.0706 | 1.216 | 0.28213 | 0.037409 |
| 2,3-Diaminopropionic acid | + | 1.183 | 105.0701 | 1.2265 | 0.29453 | 0.025187 |

| | | | | | | |
|---|---|---|---|---|---|---|
| Iditol | - | 8.796 | 181.0705 | 1.2513 | 0.32346 | 0.012512 |
| 3-Methylpyrazole | + | 0.782 | 83.06102 | 1.2536 | 0.32609 | 0.0179 |
| Hexylamine | + | 8.498 | 102.1281 | 1.2727 | 0.34789 | 0.028217 |
| Diacetyl | + | 0.045 | 87.04469 | 1.2754 | 0.3509 | 0.027043 |
| Isobutylamine | + | 8.093 | 74.09717 | 1.3082 | 0.38753 | 0.025187 |
| Tropine | + | 5.94 | 142.1226 | 1.3185 | 0.39892 | 0.004157 |
| *N*,*N*-Dimethylformamide | + | 4.177 | 74.0608 | 1.3712 | 0.45546 | 0.01473 |
| 4,4'-Sulfonyldiphenol | - | 0.769 | 249.0213 | 1.4194 | 0.50532 | 0.015113 |
| 4-Hydroxy-2-quinolinecarboxylic acid | + | 11.279 | 190.0498 | 1.444 | 0.53005 | 0.030431 |
| Benzylamine | + | 0.096 | 108.0811 | 1.4535 | 0.53956 | 0.008572 |
| 6-Hydroxycaproic acid | - | 4.348 | 131.07 | 1.4589 | 0.54488 | 0.008572 |
| Morpholine | + | 15.108 | 88.07627 | 1.4807 | 0.5663 | 0.013255 |
| 6-Pentyl-2*H*-pyran-2-one | + | 10.7 | 167.1066 | 1.4897 | 0.57501 | 0.037409 |
| Aniline | + | 0.092 | 94.06563 | 1.517 | 0.60118 | 0.005078 |
| Orcinol | + | 0.014 | 125.06 | 1.5806 | 0.66045 | 0.004157 |
| 2-Hydroxy-2-methylbutyric acid | - | 3.279 | 117.0542 | 1.7003 | 0.76583 | 0.009049 |
| Nicotine | + | 9.099 | 163.1228 | 1.913 | 0.93582 | 0.027043 |
| FA 16:3 | - | 11.557 | 249.1854 | 1.959 | 0.97014 | 0.02113 |
| Glycerol 3-phosphate | + | 11.14 | 173.0211 | 1.9644 | 0.97407 | 0.003107 |
| FA 16:2 | - | 11.976 | 251.201 | 2.0015 | 1.0011 | 0.009049 |
| FA 16:4 | - | 10.874 | 247.1697 | 2.1476 | 1.1027 | 0.027707 |
| Capryloyl glycine | - | 6.329 | 200.1281 | 2.154 | 1.107 | 0.03171 |
| NAGly 10:0/10:0 | + | 11.922 | 417.3358 | 2.1855 | 1.128 | 0.019573 |
| Vanillin-4-sulfate | - | 4.702 | 230.9962 | 2.2512 | 1.1707 | 0.022241 |
| Linolenic Acid | - | 12.298 | 277.2168 | 4.1045 | 2.0372 | 0.009049 |
| Neosolaniol | + | 12.627 | 383.1671 | 38.199 | 5.2555 | 0.0179 |

FC: Fold change

**Table S4. Brain metabolites significantly different between female *db/db* and male *db/db* mice.**

| Metabolite | ESI mode | RT [min] | m/z | FC | log2(FC) | adjusted p-value |
|---|---|---|---|---|---|---|
| PC 18:1_18:2 | + | 11.261 | 784.5834 | 0.042077 | -4.5708 | 0.000575 |
| Tyr-Tyr-Tyr | + | 10.149 | 508.2126 | 0.2048 | -2.2877 | 0.040168 |
| PI 11:0 | + | 10.494 | 534.228 | 0.24895 | -2.0061 | 0.029484 |
| Ticagrelor | + | 10.658 | 523.191 | 0.2554 | -1.9692 | 0.040168 |
| PI 14:0 | + | 10.569 | 576.2742 | 0.25772 | -1.9562 | 0.020368 |
| Neosolaniol | + | 12.627 | 383.1671 | 0.41593 | -1.2656 | 0.020368 |
| 6-Hydroxycaproic acid | - | 4.348 | 131.07 | 0.41809 | -1.2581 | 0.006379 |
| Maytenin | + | 11.444 | 421.271 | 0.48304 | -1.0498 | 0.020368 |
| Docosatrienoic acid | - | 14.312 | 333.2797 | 0.56575 | -0.82175 | 0.02829 |
| Lauroylcarnitine | - | 11.607 | 342.2642 | 0.6408 | -0.64206 | 0.020368 |
| Amphetamine | + | 3.25 | 136.1121 | 0.6515 | -0.61817 | 0.000356 |
| Muscone | + | 12.135 | 239.2369 | 0.65944 | -0.60068 | 0.020924 |
| Pristimerin | + | 11.552 | 487.2793 | 0.6609 | -0.5975 | 0.028808 |
| Adipate | + | 12.911 | 147.0654 | 0.66655 | -0.58521 | 0.036796 |
| Camphor | + | 12.882 | 153.1272 | 0.68546 | -0.54485 | 0.036796 |
| Elaidic acid | + | 12.135 | 283.2629 | 0.70435 | -0.50564 | 0.020368 |
| Batyl Alcohol | + | 11.469 | 367.3204 | 0.72597 | -0.46202 | 0.00118 |
| LPC O-18:2 | + | 11.544 | 506.3601 | 0.74841 | -0.4181 | 0.020368 |
| PA 22:3; PA 6:0-16:3 | - | 7.909 | 501.257 | 0.80341 | -0.31579 | 0.020368 |
| 1-Methylxanthine | + | 5.292 | 167.0553 | 0.8137 | -0.29743 | 0.036404 |
| Rhodinyl Acetate | + | 12.176 | 199.1692 | 0.82695 | -0.27414 | 0.048317 |
| 2,4-dihydroxyheptadec-16-en-1-yl acetate | + | 10.285 | 311.2577 | 0.83887 | -0.25347 | 0.002948 |
| Caprylic acid | - | 5.86 | 143.1064 | 0.84947 | -0.23536 | 0.047708 |
| 3-Hydroxypicolinic acid | - | 14.513 | 138.0182 | 0.86273 | -0.21303 | 0.020368 |
| 1,4-dihydroxyheptadec-16-en-2-yl acetate | + | 10.531 | 311.2577 | 0.86392 | -0.21104 | 0.040168 |
| 3,7-Epoxycaryophyllan-6-One | + | 3.767 | 275.1422 | 0.88006 | -0.18432 | 0.039901 |
| Carbazochrome sulfonate | + | 1.971 | 301.0585 | 0.8824 | -0.1805 | 0.039901 |
| Erythronolactone | - | 2.459 | 117.0179 | 0.89537 | -0.15944 | 0.011457 |
| Palmitic acid | - | 6.068 | 255.2324 | 0.89725 | -0.15642 | 0.028808 |
| Piperonylic Acid | - | 10.166 | 165.0182 | 0.899 | -0.1536 | 0.039901 |
| Phthalic acid | - | 6.413 | 165.0181 | 0.90846 | -0.13851 | 0.028808 |
| 9-Fluorenone | + | 7.617 | 181.0644 | 0.90906 | -0.13756 | 0.042721 |
| Sarcosine | + | 2.85 | 90.05552 | 1.1509 | 0.20271 | 0.048317 |
| Epinephrine | + | 2.854 | 184.0969 | 1.181 | 0.24004 | 0.040168 |
| 4-Nitro-*N*-phenylaniline | - | 9.176 | 213.0661 | 1.2377 | 0.30768 | 0.035449 |
| LPC O-16:1 | + | 10.592 | 480.3444 | 1.2405 | 0.31096 | 0.035449 |
| PC 12:0_12:0 | + | 10.175 | 622.4426 | 1.2719 | 0.34701 | 0.030274 |

| | | | | | | |
|---|---|---|---|---|---|---|
| Linoleic acid | - | 12.929 | 279.2324 | 1.2818 | 0.35818 | 0.033134 |
| LPE 20:3 | - | 10.163 | 502.2932 | 1.3656 | 0.44954 | 0.045678 |
| Xanthohumol | - | 12.666 | 353.1421 | 1.3737 | 0.45802 | 0.035449 |
| LPC 18:2 | + | 9.79 | 520.3393 | 1.4297 | 0.51568 | 0.009787 |
| 3-Hydroxybutyric acid | - | 1.815 | 103.0386 | 1.4981 | 0.58317 | 0.040168 |
| FA 16:2 | - | 11.976 | 251.201 | 1.5379 | 0.62096 | 0.002948 |
| tetradec-5-ynoic acid | - | 10.964 | 223.1695 | 1.551 | 0.63319 | 0.030274 |
| LPC 22:5 | - | 10.065 | 614.3456 | 1.6388 | 0.71266 | 0.028362 |
| Pefloxacin | + | 4.058 | 334.1608 | 1.6506 | 0.72296 | 0.029929 |
| NAGly 10:0/10:0 | + | 11.922 | 417.3358 | 1.7037 | 0.76866 | 0.036796 |
| Glucosamine 6-phosphate | + | 10.93 | 260.0527 | 1.7966 | 0.84528 | 0.030274 |
| Phosphorylcholine | + | 10.808 | 184.0736 | 1.9453 | 0.95997 | 0.02667 |
| Linolenic Acid | - | 12.298 | 277.2168 | 2.1659 | 1.115 | 0.011457 |
| PC 19:2_19:2 | + | 11.282 | 810.5973 | 2.1771 | 1.1224 | 0.035449 |
| 14-Benzoyltalatizamine | + | 9.675 | 526.313 | 2.5241 | 1.3358 | 0.035449 |
| PE 40:6; PE 20:3-20:3 | + | 14.493 | 792.5511 | 24.877 | 4.6367 | 0.035449 |
| Sphingomyelin | + | 10.921 | 731.6034 | 27.358 | 4.7739 | 0.002948 |

FC: Fold Change

**Table S5. Brain metabolites significantly different between female WT and male WT mice.**

| Metabolite | ESI | RT [min] | m/z | FC | log2(FC) | adjusted p-value |
|---|---|---|---|---|---|---|
| 2-Hydroxy-2-methylbutyric acid | - | 3.279 | 117.0542 | 0.32518 | -1.6207 | 0.002828 |
| 6-Hydroxycaproic acid | - | 4.348 | 131.07 | 0.3475 | -1.5249 | 0.002828 |
| Glycerol 3-phosphate | + | 11.14 | 173.0211 | 0.63211 | -0.66175 | 0.014628 |
| Aniline | + | 0.092 | 94.06563 | 0.77823 | -0.36174 | 0.00773 |
| Allose | - | 6.071 | 161.0443 | 0.83312 | -0.26341 | 0.012909 |
| Isovaleric acid | + | 2.849 | 103.0758 | 1.4037 | 0.4892 | 0.00773 |
| Methylsuccinic acid | - | 2.194 | 131.0336 | 1.4585 | 0.5445 | 0.041096 |
| 4-Methoxyaniline | + | 2.852 | 124.0759 | 1.5192 | 0.60335 | 0.005791 |
| 2-Phenylphenate tetrahydrate | + | 4.206 | 171.0803 | 1.542 | 0.62477 | 0.012909 |
| Ethosuximide | + | 2.853 | 142.0863 | 1.5469 | 0.62939 | 0.002828 |
| Epinephrine | + | 2.854 | 184.0969 | 1.5572 | 0.63898 | 0.00773 |
| Sarcosine | + | 2.85 | 90.05552 | 1.5943 | 0.67288 | 0.005791 |
| Betonicine | + | 2.853 | 160.0969 | 1.6318 | 0.70642 | 0.012909 |
| Pantothenic acid | - | 2.84 | 218.1025 | 1.6472 | 0.72003 | 0.00773 |
| Olmesartan | - | 2.84 | 445.1991 | 2.1675 | 1.116 | 0.032185 |

FC: Fold change
